# The influence of attractor stability of intrinsic coordination patterns on the adaptation to new constraints

**DOI:** 10.1038/s41598-020-60066-7

**Published:** 2020-02-20

**Authors:** Kota Yamamoto, Masahiro Shinya, Kazutoshi Kudo

**Affiliations:** 1 Department of Human Sciences, Kanagawa University, Yokohama, 221-8686 Japan; 2 Graduate School of Integrated Arts and Science, Hiroshima University, Higashi-Hiroshima, 739-8521 Japan; 3 Department of Life Science, Graduate School of Arts and Sciences, The University of Tokyo, Meguro, 153-8902 Japan; 4 Interfaculty Initiative in Information Studies, The University of Tokyo, Bunkyo, 113-0033 Japan

**Keywords:** Human behaviour, Dynamical systems

## Abstract

In most human movement tasks, the same goal can be achieved by a diversity of coordination patterns. For instance, when learning to juggle, individuals adopt their own unique coordination patterns in the early stages of acquiring the fundamental skills of juggling. These individual differences in the learning paths lead to differences in adaptability to new constraints. However, the reason for these differences in adaptability is still unknown. To address this problem, we quantified these differences in terms of attractor stability of the coordination patterns of expert jugglers using Recurrence Quantification Analysis. Furthermore, we quantified the attractor stability of intermediate jugglers and examined adaptability in a sensorimotor synchronization task. We found differences in attractor stability among coordination patterns of expert jugglers, as well as a difference in attractor stability between intrinsic coordination patterns of intermediate jugglers. Whereas, almost no significant direct correlation between attractor stability and adaptability of intermediate jugglers was found, suggesting a difference in both attractor stability and adaptability between intrinsic coordination patterns such that the difference in attractor stability might affect adaptability to new constraints. We submit that the learning path selected by each learner in the early stages of learning plays an important role in the subsequent development of expertise.

## Introduction

Motor tasks in many sports have redundant degrees of freedom in terms of viable problem-solving methods. Therefore, there is no singular coordination pattern to best achieve the task goal. From the viewpoint of the dynamical systems approach (see^1^ for a review), the diversity in coordination patterns arises from an interaction between the redundancy of the task solution and the multiple degrees of freedom of the human body^2,3^. For example, it has been reported that diversity arises in coordination patterns and ball trajectories for hitting a specific target in an underarm throwing task^4^. Also, when hitting a ball with a cricket bat, different coordination patterns emerged depending on the type of pitch^5^, while in boxing, the variety of punches the boxer chooses depends on the distance to the opponent^6^. Thus, in sports-related tasks, various coordination patterns appear depending on the performance environment, the situation, and the performer. On the other hand, diversity also has been reported in the coordination patterns and solution strategies acquired by learners in the process of achieving the same goal^7–10^. In our previous study^11^, a longitudinal study of the learning process associated with the fundamental skills of juggling showed that the coordination patterns acquired by learners were divided into multiple attractors with a stable temporal structure. Further, the coordination pattern developed early on in the learning process was maintained in the long run^11–14^.

The motor learning process is not completed merely by achieving one goal, but involves repeated learning and adaptation aimed at gaining expertise. For example, in juggling, most novices master the three-ball cascade juggling as a fundamental skill at the beginning of the learning process. Three-ball cascade juggling is a skill in which three balls are exchanged alternately between left and right hands, and forms the basis of many juggling skills. After acquiring this fundamental skill, learners acquire additional skills, such as matching juggling patterns to music and passing balls to other performers. However, individual differences in coordination patterns like habits can affect expertise development because the intrinsic dynamics of the individual learners reflects previous learning experiences^15^, and thus the acquired coordination pattern can become an intrinsic constraint influencing the development of new individual adaptations. In other words, the intrinsic dynamics, including coordination patterns formed by past experiences, may either promote or impede adaptation. A previous study reported that there is an asymmetry in adaptability of new tasks between multiple coordination patterns acquired in previous learning experiences^16^. The study findings suggested that in a visuomotor reaching task, participants who learned by performing discrete reaching movements in a previous process showed a nearly complete transition to performing rhythmic reaching movement without hardly any transfer in the opposite direction.

Such asymmetry of adaptation also appeared in our previous study on three-ball juggling, a multi-degree-of-freedom movement task^17^. In juggling, various coordination patterns are acquired in the learning process as described above. These coordination patterns may be roughly divided into rhythmic coordination patterns and discrete coordination patterns^17^. We examined the adaptation of intermediate jugglers to juggling in context of in accordance with the beat of a metronome. As a result of the adaptation, participants who had a discrete pattern as an intrinsic pattern showed higher adaptability than participants who had a rhythmic pattern as an intrinsic pattern. In other words, the coordination patterns acquired in a previous learning process constituted an intrinsic constraint that determined the adaptation process^17^. Therefore, in this study, we investigated the factor that leads to asymmetry in adaptability in juggling. In particular, we focused on the differences in attractor stability between different coordination patterns of motor primitives.

In the dynamical systems approach, the pattern formation of human movement is understood in terms of the concept of self-organization as advanced in non-linear dynamics and physical theories of complexity^18–20^. This approach captures human movement as a system and adopts a description of the movement in terms of “attractors,” the specific stable states to which movement trajectories converge over time^19^. An attractor is a region into which a specific trajectory or a fixed point is naturally drawn, converges, and settles down, that does not collapse when a disturbance occurs, and that can converge to the original trajectory or point^19^. Therefore, the attractors play an essential role in generating stable movements. From this, it is possible to evaluate the individual skills and their stability and stationarity by evaluating the stability of the state of the converged attractor. Descriptions and quantifications of attractors from a dynamical systems perspective have focused predominantly on a bimanual coordination task in which the left and right fingers or arms are treated as two oscillators whose relative phase describes the attractors of bimanual coordination. In-phase, in which the relative phase is 0°, or anti-phase, in which the relative phase is 180°, are the intrinsic attractors of this task^18^. It is widely known that stable performance can be achieved in the vicinity of such an inherent attractor even if some perturbation occurs.

The concept of attractor also refers to the motor learning process. In the early stages of the learning process, participants have considerable problems with producing the to-be-learned, intrinsically unstable pattern and tend to fall back into the intrinsically stable in- or anti-phase mode^21–26^. Therefore, participants have to overcome the tendency of attraction toward a 0° or 180° pattern to establish the new coordination pattern^25,27^. Nevertheless, it turned out to be possible to acquire a phase shift pattern of 90° or 45° through practice. In other words, the motor learning process is described dynamically as overcoming intrinsic constraints (attraction to stable attractors) and acquiring new coordination patterns^25,28,29^. Within this theoretical framework, some studies have examined how intrinsic attractors or pre-existing patterns affect the acquisition of new coordination patterns. For example, a strong attraction interferes with learning if the pattern to be learned is near an attractor with higher stability^26^. Some studies have provided evidence that the pattern close to the 0° attractor is performed better than that close to the 180° attractor^30,31^. That is, some studies have suggested that patterns that intrinsically stable coordination patterns before learning affect the adaptation to new constraints. However, most of the motor tasks used in these studies have less degree of freedom, so there are no large individual differences of attractors. In the present study, we investigated the stability of attractors for various coordination patterns acquired in the process of learning to juggle and examined the influence of individual differences in acquired patterns on the adaptation process.

In previous studies, descriptions and quantification of attractors were evaluated using nonlinear time series analysis^20,32–37^. In this study, we evaluated the attractor stability using Recurrence Quantification Analysis (RQA) in perspective of the degree of recurrence, determinism, and strength of attraction. In RQA, the concept of “recurrence” means that for a particular point of an attractor reconstructed in topological space, after a certain period another point on the trajectory falls close to the point in question. In RQA, it is possible to identify the essential characteristics of the deterministic dynamical system and the stability of the attractor by evaluating the recurrence plot created based on the reconstructed attractor trajectory^38,39^. In particular, we evaluated the state of the system of juggling by determining the recurrence rate, the determined rate, and Maxline. The recurrence rate for attractor fluctuation was the ratio of the number of actual recurrence points among all possible recurrence points. A smaller recurrence rate indicated more significant fluctuations in the system. The determined rate was the proportion of recurrence points that formed a diagonal structure on the recurrence plot, indicating the degree to which the attractor trajectory of the system had a deterministic structure^36^. Finally, Maxline was the maximum length of the recurrence points that continuously formed a diagonal structure. This index represented the strength of the attractor, which indicated resistance to disturbances of the system due to the strength of attraction^36,40^. In other words, RQA is a method of evaluating attractor stability of a system from the viewpoint of the degrees of recurrence, determinism structure, and strength of attraction. Recent studies have evaluated the stability and robustness of a variety of systems through the development of a description of movement in the dynamical systems approach (e.g., postural control^41^, heart rate^39,42^, inter-limb coordination^37,40^, inter-personal coordination^43,44^, and postural control of standing of ballet dancers^45^). All of these previous studies have successfully evaluated the stability of human movement using RQA.

In this paper, we applied RQA to evaluate the stability of the system for various coordination patterns of juggling and to examine the relationship with asymmetric adaptability. First, we described the differences in attractor stability for various coordination patterns appearing in the movement frequencies of expert jugglers. We also examined the influence of attractor stability on the adaptation process by comparing the performance of the adaptation task with the stability of intrinsic coordination patterns. Consequently, a difference was found in the degree of deterministic structure of attractors and strength of attraction among various coordination patterns observed in expert jugglers. Furthermore, in intermediate jugglers, attractors of discrete coordination patterns, which have higher adaptability, showed a lower degree of deterministic structure than did those of rhythmic coordination patterns. Thus, there was a difference in the stability or strength of attractors among the various coordination patterns, even though they may result in the same performance. Furthermore, this difference should be one of the factors determining adaptability. That is, an individual’s intrinsic coordination patterns acquired in the early stages of learning might play an important role in the subsequent development of expertise.

## Results

### Differences in stability across coordination patterns

**Figure 1:**
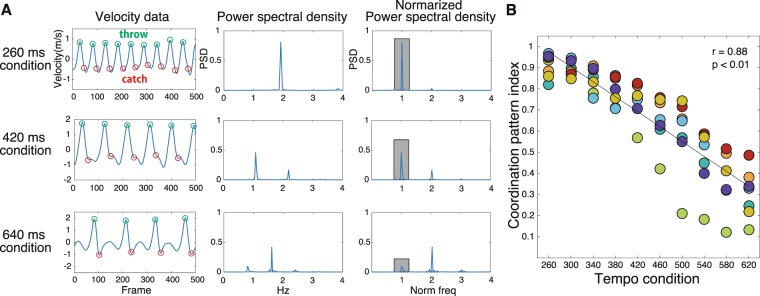
A typical example of a change in coordination pattern with a change in juggling tempo. (**A**) The vertical velocity of wrist movement with throw (green circle) and catch (red circle) timing (left column), power spectral density (middle column), and power spectrum density normalised with the tempo provided by the metronome (right column) under the 260  ms, 460  ms, and 620  ms conditions. (**B**) Analysis of the correlation between the tempo conditions and Coordination Pattern Index, calculated for all the frequency components of the normalised spectral density over an interval of 10% of the fundamental frequency component (gray square areas in figure A). The colour of the circle indicates the respective participant. The rhythmic coordination patterns with a higher ratio of the fundamental frequency component appeared when the tempo was faster, while a discrete coordination pattern with a lower ratio appeared when the tempo was slower.

In Experiment 1, we compared the RQA indices of the attractors of various coordination patterns appearing under the 10-step tempo conditions in expert jugglers. The coordination pattern of juggling under each tempo condition was described by a frequency analysis of the wrist movement in the vertical direction (Fig. 1A, modified from^16^). Under a condition with a relatively fast tempo (260  ms), the hand velocity pattern showed a sine-curve-like smooth waveform. On the other hand, under a condition with a relatively slow tempo (620  ms), the velocity pattern of the hand movement showed a waveform with a period of stopping in between velocity peaks and a high-frequency period of movement. The Coordination Pattern Index, defined as the proportion of the fundamental frequency component, showed a higher value as the tempo increased and a lower value as the tempo decreased (*n* = 70, *r* = −0.88, *p* = 0.001, mean of individual *Fisher’s z* = −0.28, 95% confidence intervals = [−0.99, −0.85], Fig. 1B).

**Figure 2:**
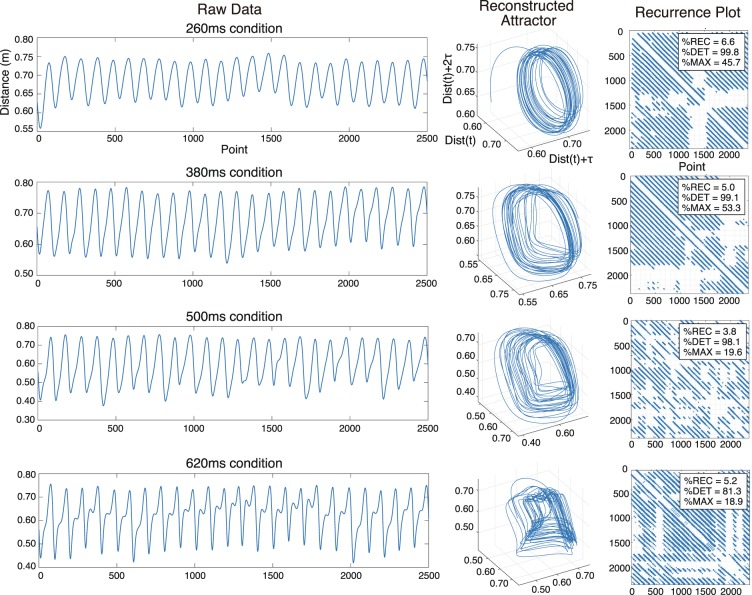
Recurrence quantification analysis of juggling movement. The raw data of distance between the vertex of the head and wrist (right column), reconstructed attractors in three-dimensional state space (middle column), and the recurrence plot (left column) in each condition, 260, 380, 500, and 620  ms. In the recurrence plot, the recurrence rate (%REC), the determined rate (%DET), and the maximum line length ratio (%MAXLINE) of each set of data are described. Although the actual embedment dimension used in this study is six dimensions, we show the attractor trajectory reconstructed in three dimensions for ease of visualisation.

Figure 2 shows a typical example of RQA, it presents the raw data of a three-directional distance between the vertex of the head and wrist, reconstructed attractors in three-dimensional state space, and a recurrence plot for each condition (260, 380, 500, and 620  ms). In the recurrence plot, the recurrence rate (%REC), the determined rate (%DET), and the maximum line length ratio (%MAXLINE) for each set of data are depicted. Although the actual embedding dimension used in this study is six, an attractor trajectory reconstructed in three dimensions is shown for visualization. For instance, a reconstructed attractor in the 260  ms condition (top row) showed a stable trajectory, and thus more recurrent points and a longer diagonal structure in the recurrence plot (right column) than the attractor and recurrence plot for the 500  ms or 620  ms condition. This visual difference in attractor trajectory or recurrence plot corresponded to the difference in %REC, %DET, or %MAXLINE for each condition (%REC = 6.6, %DET = 99.8, and %MAXLINE = 45.7 for the 260  ms condition versus %REC = 3.8, %DET = 98.1, and %MAXLINE = 19.6 for the 500  ms condition).

**Figure 3:**
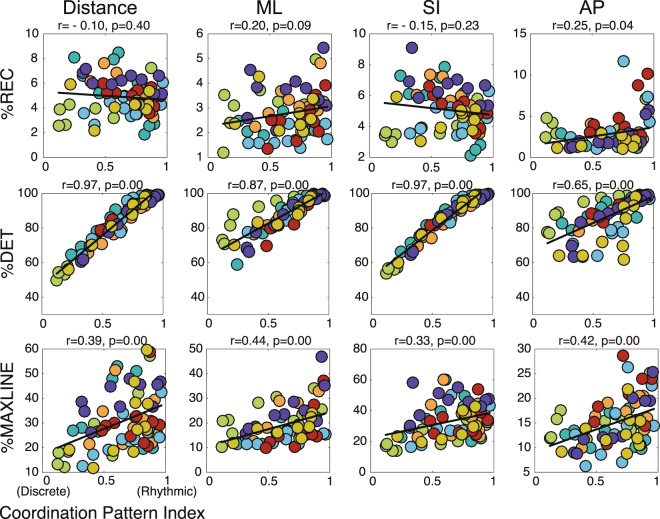
Difference in stability and strength of attractors across coordination patterns in expert jugglers. The figure shows the correlation between Coordination Pattern Index and %REC (top row), %DET (middle row), and %MAXLINE (bottom row) of three-dimensional distance, ML, SI, and AP directional movement of expert jugglers in Experiment 1. The colour of the circle indicates the respective participant. In all figures, the *r* and *p* values represent the strength and significance of the Pearson correlation coefficients between the variables of interest. There was no correlation between the Coordination Pattern Index and the %REC. In contrast, there was a strong correlation between Coordination Pattern Index and %DET, and a moderate correlation was found with %MAXLINE. These results mean that the attractor of the rhythmic coordination pattern is more stable and stronger than the attractor of the discrete coordination pattern.

Figure 3 shows the results of a correlation analysis using the Coordination Pattern Index of multiple juggling patterns and corresponding RQA index. For this analysis, the significance level (α) was set to 0.41% using the Bonferroni correction to prevent the inflation of false positives. Regarding %REC, there was no correlation between the Coordination Pattern Index and %REC for 3D distance (*n* = 70, Pearson’s *r* = −0.10, *p* = 0.399, mean of individual *Fisher’s z* = −0.28, 95% confidence intervals = [−0.77, 0.42]) or hand movement in the mediolateral direction (*n* = 70, *r* = 0.20, *p* = 0.090, mean *z* = 0.40, *CI* = [−0.32, 0.81]), superoinferior direction (*n* = 70, *r* = −0.15, *p* = 0.229, mean *z* = −0.33, *CI* = [−0.79, 0.39]), or anteroposterior direction (*n* = 70, *r* = 0.25, *p* = 0.036, mean *z* = 0.25, *CI* = [−0.46, 0.76]). That is, the attractors of the rhythmic and discrete coordination patterns had a similar degree of recurrence. This result indicates that the attractors of both coordination patterns had sufficient stability against fluctuations to allow for continued juggling. On the other hand, with respect to the %DET, there were strong correlations between the Coordination Pattern Index and %DET for 3D distance (*n* = 70, *r* = 0.97, *p* < 0.001, mean *z* = 2.54, *CI* = [0.95, 0.99]), as well as hand movement in the mediolateral direction (*r* = 0.87, *p* < 0.001, mean *z* = 1.99, *CI* = [0.85, 0.99]), superoinferior direction (*n* = 70, r = 0.97, *p* < 0.001, mean *z* = 2.24, *CI* = [0.93, 0.99]), and anteroposterior direction (*n* = 70, *r* = 0.65, *p* < 0.001, mean *z* = 1.50, *CI* = [0.64, 0.98]). This result implies that the attractor trajectory of the rhythmic coordination pattern had a higher deterministic structure than that of the discrete coordination pattern. As for %MAXLINE, there were weak correlations between the Coordination Pattern Index and the %MAXLINE for 3D distance (*n* = 70, *r* = 0.39, *p* < 0.001, mean *z* = 0.49, *CI* = [−0.25, 0.84]), as well as hand movement in the mediolateral direction (*n* = 70, *r* = 0.44, *p* < 0.001, mean *z* = 0.82, *CI* = [0.08, 0.92]), the superoinferior direction (*n* = 70, *r* = 0.33, *p* < 0.001, *mean z* = 0.40, *CI* = [−0.33, 0.81]), and the anteroposterior direction (*n* = 70, *r* = 0.42, *p* < 0.001, mean *z* = 0.51, *CI* = [−0.22, 0.85]). That is, the rhythmic coordination pattern showed an attractor strength relatively higher than that of the discrete coordination pattern, indicating that while any juggling pattern had a degree of recurrence sufficient to continue juggling, rhythmic patterns had a higher degree of deterministic structure and strong attraction than discrete patterns.

### Differences in attractor stability among intrinsic coordination patterns for intermediate jugglers

In Experiment 2, we examined the effect of attractor stability on adaptability to sensorimotor synchronization tasks in intermediate jugglers. Our previous study^16^ showed that the intermediate jugglers who had intrinsically discrete coordination patterns showed greater adaptability to the sensorimotor synchronization task than those who had intrinsically rhythmic coordination patterns. By integrating the results on adaptability to new constraints for the intermediate jugglers and the results on differences in attractor stability between the coordination patterns of the expert jugglers in the previous section, it was hypothesized that participants who had attractors with low stability would be more likely to adapt to new constraints. Therefore, we examined the relationship between the intrinsic coordination pattern of the intermediate jugglers and the RQA indices, as well as the relationship between the RQA indices and the performance on the adaptation task.

**Figure 4:**
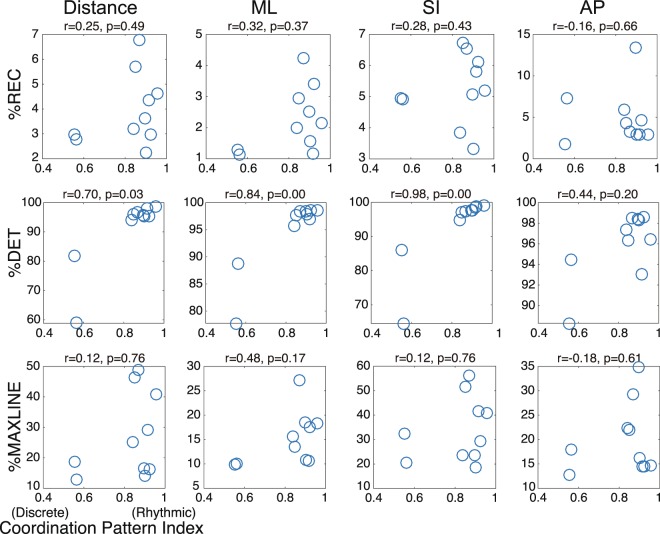
Difference in stability and strength of attractors across coordination patterns in intermediate jugglers. The figure shows the correlation between Coordination Pattern Index and %REC (top row), %DET (middle row), and %MAXLINE (bottom row) for three-dimensional distance and the ML, SI, and AP directional movements of intermediate juggles in Experiment 2. In all figures, *r* and *p* values represent the strength and significance of the Spearman correlation coefficients between the variables of interest. There was no correlation between the Coordination Pattern Index and %REC and %MAXLINE. In contrast, a strong correlation was found for %DET. These results indicate that the attractor of the rhythmic coordination pattern is more stable than the attractor of the discrete coordination pattern.

First, we examined the relationship between the coordination pattern and corresponding RQA index. The coordination pattern of each participant was such that when juggling without the tempo being set by the metronome, the participants should have displayed an intrinsic coordination pattern. Figure 4 shows the results of the correlation analysis for the intermediate jugglers of the Coordination Pattern Index when juggling at the preferred tempo and each RQA index. Before the correlation analysis, we examined whether the data were distributed normally. A normal data distribution was not confirmed, so we calculated the correlation coefficient using Spearman’s correlation. In this analysis, the significance level (α) was also set to 0.41% using the Bonferroni correction. The %REC showed no correlation with the preferred coordination pattern (for %REC and Coordination Pattern Index in the mediolateral direction, *n* = 10, Spearman’s *r* = 0.32, p = 0.365; in the superoinferior direction, *n* = 10, *r* = 0.28, p = 0.425; in the anteroposterior direction, *n* = 10, *r* = −0.16, p = 0.651; or for 3D distance, *n* = 10, *r* = 0.25, p = 0.489). This means that there was no significant difference in the degree of recurrence between rhythmic and discrete coordination patterns. For %DET, strong correlations were found between the Coordination Pattern Index and %DET for the hand movement in the mediolateral direction (*n* = 10, *r* = 0.84, *p* = 0.002) and the superoinferior direction (*n* = 10, *r* = 0.98, *p* < 0.001), but not in the anteroposterior direction (*n* = 10, *r* = 0.44, *p* = 0.200) and 3D distance (*n* = 10, *r* = 0.70, *p* = 0.025). This means that the attractor trajectory of the rhythmic coordination pattern had a higher degree of deterministic structure than the discrete pattern. As for %MAXLINE, there was no correlation with the preferred coordination pattern (for %MAXLINE and Coordination Pattern Index in the mediolateral direction, *n* = 10, *r* = 0.48, p = 0.162.; in the superoinferior direction, *n* = 10, *r* = 0.12, p = 0.751; in the anteroposterior direction, *n* = 10, *r* = −0.18, p = 0.614; or for 3D distance, *n* = 10, *r* = 0.12, p = 0.751).

### The relationship between adaptability and stability of attractors

**Figure 5:**
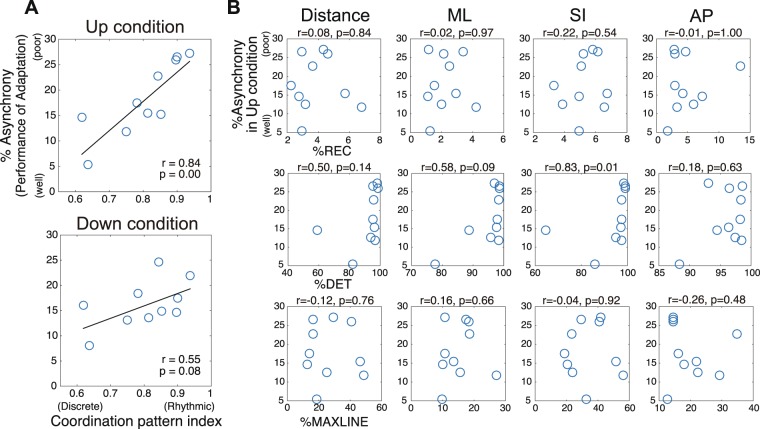
Performance of the adaptation task for various coordination patterns and the influence of attractor stability on adaptability. (**A**) Pearson’s correlation coefficients between intrinsic coordination pattern (Coordination Pattern Index) and performance on the adaptation task (%Asynchrony, the normalised absolute error between the beep and catch timing) for intermediate jugglers. (**B**) Spearman’s correlation coefficients between %Asynchrony and %REC (top row), %DET (middle row), and %MAXLINE (bottom row). There was a significant correlation between %Asynchrony and %DET for distance and movement in the SI direction. However, there was no significant correlation with the other variables.

Next, we examined the relationship between the RQA indices and the performance on adaptation tasks. As an adaptation task for intermediate jugglers who could perform three-ball juggling, we set up a sensorimotor synchronization task for them to juggle while timing their catch to metronome beeps which either increased or decreased in tempo. To measure the performance of this adaptation task, we calculated %Asynchrony, which is the normalized absolute error between the beep and catch timing by metronome interval. %Asynchrony was obtained by scaling asynchrony based on the tempo-beep interval when an error occurred. This provided a ratio of errors for the tempo-beep interval. Figure 5A shows the results of a correlation analysis between %Asynchrony in the Up condition, in which the tempo increased, in the Down condition, in which the tempo decreased, and the Coordination Pattern Index at free tempo juggling, which was used to indicate the participants’ intrinsic coordination patterns. For this analysis, the significance level (α) was set to 2.5% using the Bonferroni correction. There was a strong correlation and correlation trend between performance on the adaptation task and intrinsic coordination patterns in the Up and in the Down conditions, respectively (Up: *n* = 10, *r* = 0.84, *p* = 0.002, Down: *n* = 10, *r* = 0.55, *p* = 0.06).

Figure 5B shows the results of the correlation analysis between %Asynchrony in the Up condition and in the Down condition, and the RQA indices. For this analysis, the significance level (α) was set to 0.41% using the Bonferroni correction. Regarding %REC, there was no correlation between %Asynchrony and %REC for hand movement in either condition for 3D distance (Up: *n* = 10, *r* = 0.08, *p* = 0.829, Down: *n* = 10, *r* = −0.15, *p* = 0.676), the mediolateral direction (Up: *n* = 10, *r* = 0.02, *p* = 0.960, Down: *n* = 10, *r* = −0.10, *p* = 0.777), the superoinferior direction (Up: *n* = 10, *r* = 0.22, *p* = 0.533, Down: *n* = 10, *r* = −0.03, *p* = 0.934), or the anteroposterior direction (Up: *n* = 10, *r* = −0.01, *p* = 0.987, Down: *n* = 10, *r* = 0.26, *p* = 0.467). On the other hand, there was a correlation between %Asynchrony in the Up condition and %DET in the superoinferior direction (*n* = 10, *r* = 0.83, *p* = 0.003). However, there was no correlation between %Asynchrony in the Up condition and %DET in either the mediolateral direction (*n* = 10, *r* = 0.58, *p* = 0.08), the anteroposterior direction (*n* = 10, *r* = 0.18, *p* = 0.627) or 3D distance (*n* = 10, *r* = 0.50, *p* = 0.138). Furthermore, there was no correlation between %Asynchrony in the Down condition and %DET for 3D distance (*n* = 10, *r* = 0.27, *p* = 0.446), the mediolateral direction (*n* = 10, *r* = 0.42, *p* = 0.229), the superoinferior direction (*n* = 10, *r* = 0.55, *p* = 0.098), or the anteroposterior direction (*n* = 10, *r* = 0.25, *p* = 0.489). This indicates that performance on the adaptation task is related to the deterministic structure of the attractor trajectory for hand movement. There was also no statistically significant correlation between %Asynchrony in the Up and in the Down conditions and %MAXLINE for hand movement in the mediolateral direction (Up: *n* = 10, *r* = 0.16, *p* = 0.651, Down: *n* = 10, *r* = 0.13, *p* = 0.726), the superoinferior direction (Up: *n* = 10, *r* = −0.04, *p* = 0.907, Down: *n* = 10, *r* = −0.37, *p* = 0.293), the anteroposterior direction (Up: *n* = 10, *r* = −0.26, *p* = 0.476, Down: *n* = 10, *r* = 0.07, *p* = 0.854), or 3D distance (Up: *n* = 10, *r* = −0.12, *p* = 0.751, Down: *n* = 10, *r* = −0.41, *p* = 0.244). In other words, it was shown that %MAXLINE indicating attractor strength is not directly related to adaptive performance.

## Discussion

In this study, we examined the stability of attractors for various coordination patterns that can appear in juggling tasks and the influence of those differences in attractor stability on the adaptability to new constraints. Based on an examination of coordination patterns produced by expert and intermediate jugglers, there was a difference in attractor stability between the discrete and rhythmic coordination patterns, in that the rhythmic coordination pattern had higher determinism of attractor trajectory and strength of attraction than the discrete coordination pattern. In other words, there was a difference in system stability or regularity between the coordination patterns that may be employed to continue three-ball juggling. Also, our previous work showed that intermediate jugglers with discrete coordination patterns show grater adaptability to sensorimotor synchronization tasks than those with rhythmic coordination patterns. By combining the results of this previous study with data from experts in the present study, it was hypothesized that higher attractor stability or robustness would be related to poor adaptability. Moreover, the relationship between the preferred coordination patterns of intermediate jugglers and RQA indices was similar to the results for the expert jugglers. In addition, we examined the relationship between performance on an adaptation task and the RQA indices and found that the relation with adaptation performance related to the determined rate, where there was a strong correlation with the difference in stability among coordination patterns. However, there was no direct relationship with adaptation performance for the other indices.

In particular, for RQA, there was a marked difference in the determined rate among various coordination patterns in juggling. The determined rate indicates the degree of the deterministic structure of the attractor trajectory of a system, and it was shown that the rhythmic coordination pattern had a higher degree of deterministic structure than the discrete coordination pattern. A system with a high degree of deterministic structure means that states occurring later are more closely determined by the previous states or history of the system^41^. In other words, a system with high deterministic structure has correspondingly high predictability of future states or regularity of the system. In addition, hand trajectory for the rhythmic coordination pattern fluctuated around the stable attractor and moved periodically and stably. This means that the attractor of a system with a high degree of determinism would be a stable attractor. On the other hand, the discrete coordination pattern had a less deterministic structure than the rhythmic coordination pattern. It included not only the fluctuations around the attractor, but also phases that moved out of the trajectory. Differences in attractor stability may reflect qualitative differences in movement. Rhythmic coordination patterns and discrete coordination patterns are different in movement frequency and temporal structure.

This deviated phase is thought to have occurred in the transition from catching a ball to throwing a ball. In particular, the discrete coordination pattern was composed of a series of movements divided by each ball catch event. At this time, there was a period of waiting for the ball to be thrown and fall from the opposite hand, and it was conceivable that the movement of the hand, dependent on the event timing of the opposite hand, destroyed the deterministic structure. The difference in the deterministic structure of attractors was also reflected in some differences in the Maxline index indicating the attractor strength. However, especially for the intermediate group in Experiment 2, the sample size was small and normality was not confirmed. Therefore, in Experiment 2, significance was tested using a nonparametric method. In order to confirm the validity of the results, it is necessary to acquire additional data on more intermediate jugglers and jugglers with various skill levels in future studies.

Attractors in motor control systems typically play an important role in supporting stable performance^20,25^. In the juggling task, one of these multiple attractors was acquired early in the process of learning the fundamental skills of juggling^12^. In particular, it was crucial for jugglers to control the timing of events such as throws and catches, and that these multiple attractors of coordination patterns had stability in the temporal structure of juggling. In other words, stable attractors with a stable temporal structure can support stable juggling performance. In juggling, it is not easy to repeat a stable ball trajectory because it is necessary to throw and catch the ball continuously while controlling both hands at the same time^46^. Therefore, although the height and position of the thrown ball show variability, for juggling to continue, the regular trajectories of the hands and balls have to be maintained to a certain degree. It is the attraction to the attractor that generates such consistency. However, adaptation to new sensorimotor tasks showed a poorer performance for rhythmic coordination patterns than discrete ones^17^. In this adaptation process, it was necessary to perform juggling following a gradually changing tempo, which required changes in coordination. However, attractors are characterized as attraction of stable movements to maintain robust performance against variability^18^. Therefore, it may be difficult to change from an intrinsically stable movement to a to-be-learned movement trajectory or coordination pattern. The results of this study showed that a discrete coordination pattern with good adaptive performance was lower in system stability, which is the degree of deterministic structure, and attractor strength. In other words, the stability or robustness of the attractor can be one of the factors that determines adaptability to new constraints.

On the other hand, differences in coordination patterns and stability of attractors seen in juggling can be considered from the viewpoint of motor primitives. The most basic movement component is called a motor primitive, and discrete movements such as reaching movements and rhythmic movements, like periodic motions, are the basic movement primitives of human movement^47^. As mentioned above, there was an asymmetric transfer between rhythmic and discrete movement^16^. This asymmetry of transfer has been attributed to the fact that rhythmic motor primitives include some discrete motor primitives^48,49^. That is, it is possible that the two motor primitives are not entirely independent and that some of the rhythmic motor primitives may be contained in the sequence of discrete motor primitives^16,50–53^. Based on the evidence from studies on controlled movement, it is conceivable that movement controlled by rhythmic motor primitives is controlled more simply than by discrete motor primitives. In other words, it is suggested that the rhythmic juggling movement produces consistency and stability of movement by maintaining a constant tempo involving relatively simple control. Conversely, the discrete juggling pattern generates a series of motions involving more complicated control than the rhythmic coordination pattern, and stability may be created by controlling the timing based on information on the position or velocity of the ball. For this reason, it is difficult for rhythmic coordination patterns to respond flexibly to sudden disturbances and inputs, and as a result, their adaptability in the sensorimotor synchronization task might be poorer than for the discrete pattern.

In this study, we investigated the relationship between attractor stability and adaptability for multiple movements to achieve a specific task goal. Future work should examine whether the present results may be generalized to toher motor tasks, which we expect to be the case. Likewise, we expect that the concept of movement primitives may be generalized to various movements with rhythm. To confirm these expectations, it is necessary to examine whether similar results can be obtained for a range of motor tasks that have various solutions among individuals.

In conclusion, in juggling, there are multiple coordination patterns with different control strategies. These coordination patterns are acquired early in the process of learning fundamental skills and can become habits for learners. The difference in the stability of these coordination patterns as a system relate to adaptability, which requires flexibility. Our findings highlight the importance of taking into account the diversity of learning paths in the study of complex motor learning processes.

## Methods

### Participants and protocol

For Experiment 1, seven expert jugglers (males, age 19.5 ± 0.5 years) who could perform five- or seven-ball juggling participated in the study. The definition of expert juggler was based on previous research^53^. Participants were asked to perform juggling under 10 conditions with metronome beep intervals of 260, 300, 340, 380, 420, 460, 500, 540, 580, and 620  ms. Each tempo condition lasted for 65 beeps. Participants were required to perform three-ball cascade juggling while adjusting the catch timing to the metronome beep timing (created by Audacity version 2.1.2.0, http://audacity.sourceforge.net/). We did not instruct participants about the coordination patterns in each condition. Participants were asked to adjust their catch timing as soon as possible after the beeps began.

For Experiment 2, ten intermediate jugglers (eight males and two females, age 20.6 ± 2.7 years) who could perform three-ball juggling participated in the study. We established an adaptation task with two conditions in which the participants juggled following an auditory metronome whose tempo gradually changed. In the Up condition, the interval of the beeps gradually increased from 600  ms to 300  ms in intervals of 3  ms, while the inverse occurred in the Down condition. One trial consisted of 101 beeps. In order to verify the intrinsic pattern of each participant, participants performed juggling without the metronome sound for 30  seconds as the Preferred condition. The instructions were the same as in Experiment 1. Three trials were performed under each condition.

The study was carried out in accordance with the approved guidelines and approved by the Ethics Committee of the Graduate School of Arts and Sciences, The University of Tokyo. All participants gave their written informed consent to participate.

### Data collection

An optical motion capture system with four cameras (100  Hz, Optitrack, Natural Points) was used to record the participants’ movements during all trials. Three balls (6.6  cm in diameter and mass 130  g) were covered with reflective tape. The cameras were placed around the participant so that the participant and the balls being juggled were all in view. The three-dimensional coordinates of the markers (x-axis: anterior-posterior, y-axis: vertical, z-axis: lateral-medial) were calculated using Motive software. Reconstruction of the known marker positions on the calibration frame before each learning session yielded residual errors of reconstruction of less than 1  mm for each coordinate. Also, the metronome sound generated by the PC was recorded by a data acquisition device (1000  Hz, NI-USB 6218, National Instrument) and recorded by Labview (National Instrument).

### Data reduction

The obtained metronome sound data and the synchronization signal (1000  Hz) were down-sampled to 100  Hz by the thinning method to synchronize with the motion data of Optitrack. The digitized coordinates of the three balls were identified and tracked using Motive. Missing data points were interpolated automatically by the spline method using Motive. The raw displacement data were filtered using a second-order Butterworth digital filter for each marker, with a cutoff frequency defined using residual analysis^54^. The filtered displacement values along the y-axis were differentiated to obtain the velocity of the ball and hand movement in the vertical direction. The velocity profile of the hand was used to describe the movement pattern during juggling. For RQA, we calculated the three-dimensional distance between the vertex of the head and the wrist, as well as the position data of the wrist movement in the M ediolateral (ML), Superoinferior (SI), and Anteroposterior (AP) directions.

### Coordination pattern index

In Experiments 1 and 2, the differences in the coordination patterns of juggling were described using frequency analysis. The frequency characteristics were analyzed by calculating the power spectral density after a Fourier transformation of the velocity data of vertical hand movement. For data from the expert group in Experiment 1, we calculated the spectral density of the vertical velocity of the hand under each tempo condition, and the frequency component provided by the metronome was normalized as the fundamental frequency component. The proportion of the fundamental frequency component was calculated within a range of 10%, and defined as the Coordination Pattern Index (Fig. 1A). For data from the intermediate jugglers in Experiment 2, the Coordination Pattern Index for juggling in the Preferred condition was calculated to describe the intrinsic coordination pattern. In both Experiments 1 and 2, the values of the Coordination Pattern Index of the left and right hands were averaged and used as the representative value for each participant.

### Recurrence quantification analysis

In Experiment 1, data on the position of the wrist during 65 cycles was obtained under 10 tempo conditions, of which 25 cycles each of the right and left hands were analyzed. The three-dimensional distance between the vertex of the head and wrist and the position data for the ML, SI, and AP directions of wrist movement for each tempo condition were standardized to 2500 points. In Experiment 2, we also analyzed 25 cycles each of the right and left hands while juggling at a free tempo for 30  seconds. Similarly, the three-dimensional distance and the position in the ML, SI, and AP directions of wrist movement were standardized to 2500 points. As mentioned above, in RQA, the concept of “recurrence” means that for a particular point of an attractor reconstructed in topological space, after a certain period another point in the trajectory falls close to the given point.

We explain the procedure of RQA below (for details, see^39^). First, in order to calculate the time delay (τ) for each dimension when reconstructing the data into a high-dimensional attractor, we calculated the average mutual information^55^. The time at which the average mutual information is minimized at the beginning was determined as the delay time (*t*) and was reconstructed shifted by *t* for each dimension of the state space. Also, we determined the embedding dimension (*m*) to reconstruct the attractor using the false nearest neighbors method^56–58^. In the present study, the delay time *t* was set at 25 frames (roughly a quarter of a cycle), and the embedding dimension *m* was set at six dimensions. Based on these two calculated parameters, each set of time series data was reconstructed as a high-dimensional attractor with time delay^59,60^.

Figure 2 shows a reconstructed attractor for the three-dimensional distance data based on these parameters. After reconstructing the attractor, we determined the other parameters for the RQA, which included a radius parameter (*e*), minimum recurrence time, and minimum line length. These parameters were thresholds for how close to each other the attractor trajectory points must be to be regarded as constituting “recurrence.” The radius parameter *e* used in this study was set at 15% of the maximum distance, and the minimum recurrence time and the minimum line length were set at 10 points.

We calculated the distance between all points of the trajectory in the attractor, then constructed a recurrence matrix based on the thresholds determined by these parameters and drew the matrix on the recurrence plot (Fig. 2, right). Based on the recurrence matrix, three variables were calculated: the recurrence rate (%REC), the determined rate (%DET), and the maximum line length rate (%MAXLINE), to evaluate the stability or predictability of the attractor. %REC indicates the proportion of points that actually recurred out of all possible recurrence points; %DET indicates the proportion of the recurred points forming the diagonal structure; and %MAXLINE shows the ratio of the maximum value of the recurrence point that actually formed the diagonal structure to the maximum possibility of the diagonal structure. In both Experiments 1 and 2, the indices of the right and left hands were averaged and used as the representative value for each participant.

### Performance of the adaptation task

In Experiment 2, to investigate whether the participants adapted to the task, we calculated %Asynchrony, the absolute error between each catch and each beep timing, which indicated the accuracy of performance of juggling in time with changing metronome beeps. %Asynchrony was obtained by scaling Asynchrony based on the tempo beep interval at which an error occurs, and indicated the ratio of errors to the tempo beep interval. We removed the first six of the 101 catches and analyzed the remaining 95 catches. For three participants, the number of catches included in one trial was small (at least 63 catches) because a ball fell during the trial. Furthermore, because the tempo gradually changed during one trial, the ratio of absolute error for the requested tempo was calculated as %Asynchrony.

### Statistical analysis

In Experiment 1, we conducted Pearson’s correlation analyses between juggling tempo and the Coordination Pattern Index during juggling under each tempo condition, and between the Coordination Pattern Index and each RQA index. In Experiment 2, application of the Shapiro-Wilk method did not indicate normality, and accordingly Spearman’s rank correlation analysis was performed. We examined the rank correlations between the Coordination Pattern Index during juggling under the free tempo condition and each RQA index. Furthermore, we analyzed the correlation between each RQA index and the Coordination Pattern Index or performance on the adaptation task (%Asynchrony). For the analyses in Figs. 1B and 3, we performed a Fisher z transformation for the individual correlation coefficient, and calculated 95% confidence intervals from the mean z-value to examine the robustness of the statistical results. Furthermore, we performed the Bonferroni correction for each analysis to prevent the inflation of false positive rates. The significance level was set at 0.41% (α = 0.0041) for analyses in Figs. 3, 4, and 5B. In addition, the statistical data used to analyze the correlations for each participant in Figs. 1B and 3 are attached in the Supplementary Information.

## Supplementary information

Supplementary tables.

## Data Availability

The datasets generated during and/or analysed during the current study are available from the corresponding author on reasonable request.
