# Supplementary material for: The influence of attractor stability of intrinsic coordination patterns on the adaptation to new constraints
Source: Sci Rep. 2020 Feb 20;10:3058. doi: 10.1038/s41598-020-60066-7 (PMC7033107; doi:10.1038/s41598-020-60066-7)
Supplement: Supplementary file 1 — Supplementary tables. [file 41598_2020_60066_MOESM1_ESM.pdf]

The influence of the stability of the attractors among intrinsic coordination patterns on adaptation to new constraints

Kota Yamamoto[1*], Masahiro Shinya[2], and Kazutoshi Kudo[3,4]

Affiliation

1 Department of Human Sciences, Kanagawa University, Yokohama, 221-8686, Japan

2 Graduate School of Integrated Arts and Science, Hiroshima University, Higashi-Hiroshima, 739-0046, Japan

3 Department of Life Science, Graduate School of Arts and Sciences, The University of Tokyo, Meguro, 153-0041, Japan

4 Graduate School of Interdisciplinary Information Studies, The University of Tokyo, Bunkyo, 113-8654, Japan

*ykota13@gmail.com

Corresponding Authors

Kota Yamamoto (Ph. D) and Kazutoshi Kudo (Ph. D)

Supplemental Table 1. Results of correlation analysis of each participant in analysis of Figure 1.

		Coordination Pattern Index						
		Expert 1	Expert 2	Expert 3	Expert 4	Expert 5	Expert 6	Expert 7
Tempo Condition	260	0.935	0.819	0.886	0.966	0.949	0.955	0.859
	300	0.871	0.897	0.882	0.946	0.870	0.933	0.848
	340	0.780	0.858	0.909	0.754	0.892	0.895	0.831
	380	0.710	0.862	0.850	0.708	0.857	0.798	0.755
	420	0.570	0.761	0.816	0.748	0.826	0.706	0.768
	460	0.422	0.610	0.735	0.655	0.759	0.627	0.750
	500	0.209	0.569	0.657	0.648	0.716	0.551	0.743
	540	0.182	0.450	0.588	0.536	0.582	0.399	0.573
	580	0.122	0.322	0.494	0.413	0.516	0.323	0.414
	620	0.135	0.246	0.383	0.328	0.485	0.340	0.219
r (pearson's r)		-0.979	-0.946	-0.952	-0.968	-0.966	-0.987	-0.883
t		-13.748	-8.282	-8.845	-10.866	-10.542	-17.202	-5.312
n		10	10	10	10	10	10	10
p_value		0.000	0.000	0.000	0.000	0.000	0.000	0.001
z_r(Fisher transformation)		-2.285	-1.795	-1.858	-2.056	-2.026	-2.505	-1.388
z_u(upper)		-1.544	-1.055	-1.117	-1.315	-1.285	-1.764	-0.647
z_l(lower)		-3.026	-2.536	-2.599	-2.796	-2.767	-3.246	-2.128
r_u (95% upper limit)		-0.913	-0.784	-0.807	-0.865	-0.858	-0.943	-0.570
r_l (95% lower limit)		-0.995	-0.988	-0.989	-0.993	-0.992	-0.997	-0.972

Supplemental Table 2. Results of correlation analysis of each participant in analysis of Figure 3.

		ML direction			SI direction			AP direction			3D distance		
iSubject	variables	%REC	%DET	%MAX	%REC	%DET	%MAX	%REC	%DET	%MAX	%REC	%DET	%MAX
Expert 1	r	0.6063	0.9060	0.6555	0.8212	0.9909	0.7799	0.1219	0.8746	0.3850	0.6466	0.9948	0.7032
	p	0.0631	0.0003	0.0396	0.0036	0.0000	0.0078	0.7373	0.0009	0.2719	0.0434	0.0000	0.0233
	z_r	0.7031	1.5047	0.7850	1.1605	2.6967	1.0452	0.1225	1.3523	0.4060	0.7694	2.9703	0.8736
	z_u	1.4439	2.2455	1.5258	1.9013	3.4375	1.7860	0.8633	2.0931	1.1468	1.5102	3.7111	1.6144
	z_l	-0.0377	0.7639	0.0442	0.4197	1.9558	0.3044	-0.6183	0.6115	-0.3348	0.0286	2.2295	0.1328
	r_u	0.8945	0.9778	0.9097	0.9563	0.9979	0.9453	0.6980	0.9700	0.8167	0.9070	0.9988	0.9238
	r_l	-0.0377	0.6434	0.0441	0.3967	0.9608	0.2953	-0.5500	0.5452	-0.3229	0.0286	0.9771	0.1320
Expert 2	r	0.2557	0.9725	0.7799	-0.7344	0.9799	-0.0543	-0.5836	0.7688	-0.0196	-0.7254	0.9825	-0.0995
	p	0.4758	0.0000	0.0078	0.0156	0.0000	0.8816	0.0765	0.0094	0.9571	0.0176	0.0000	0.7845
	z_r	0.2615	2.1373	1.0452	-0.9382	2.2949	-0.0543	-0.6679	1.0173	-0.0196	-0.9190	2.3638	-0.0998
	z_u	1.0023	2.8781	1.7860	-0.1974	3.0357	0.6865	0.0729	1.7581	0.7212	-0.1782	3.1046	0.6410
	z_l	-0.4793	1.3965	0.3044	-1.6790	1.5541	-0.7951	-1.4087	0.2765	-0.7604	-1.6598	1.6230	-0.8406
	r_u	0.7626	0.9937	0.9453	-0.1949	0.9954	0.5957	0.0728	0.9423	0.6176	-0.1763	0.9960	0.5656
	r_l	-0.4457	0.8846	0.2953	-0.9327	0.9145	-0.6613	-0.8872	0.2696	-0.6413	-0.9302	0.9251	-0.6861
Expert 3	r	0.5121	0.9936	0.5793	-0.8136	0.9941	-0.2301	0.0442	0.9909	0.5157	-0.8222	0.9922	-0.0110
	p	0.1302	0.0000	0.0792	0.0042	0.0000	0.5224	0.9036	0.0000	0.1270	0.0035	0.0000	0.9760
	z_r	0.5655	2.8732	0.6614	-1.1377	2.9083	-0.2343	0.0442	2.6918	0.5705	-1.1635	2.7693	-0.0110
	z_u	1.3063	3.6140	1.4022	-0.3969	3.6491	0.5065	0.7850	3.4326	1.3113	-0.4227	3.5101	0.7298
	z_l	-0.1753	2.1324	-0.0794	-1.8785	2.1674	-0.9751	-0.6966	1.9510	-0.1703	-1.9043	2.0285	-0.7518
	r_u	0.8633	0.9985	0.8858	-0.3773	0.9986	0.4672	0.6556	0.9979	0.8646	-0.3992	0.9982	0.6230
	r_l	-0.1735	0.9723	-0.0792	-0.9544	0.9741	-0.7510	-0.6022	0.9604	-0.1687	-0.9566	0.9660	-0.6362
Expert 4	r	0.4317	0.9179	0.6451	0.4285	0.9409	0.6477	0.5047	0.8761	0.5390	0.5422	0.9306	0.7543
	p	0.2129	0.0002	0.0440	0.2166	0.0000	0.0429	0.1369	0.0009	0.1079	0.1054	0.0001	0.0117
	z_r	0.4619	1.5757	0.7669	0.4581	1.7457	0.7714	0.5555	1.3586	0.6027	0.6072	1.6632	0.9828
	z_u	1.2027	2.3165	1.5077	1.1989	2.4865	1.5122	1.2963	2.0994	1.3435	1.3480	2.4040	1.7237
	z_l	-0.2789	0.8349	0.0261	-0.2827	1.0049	0.0306	-0.1853	0.6178	-0.1381	-0.1336	0.9224	0.2420
	r_u	0.8345	0.9807	0.9065	0.8333	0.9863	0.9073	0.8608	0.9704	0.8725	0.8736	0.9838	0.9383
	r_l	-0.2719	0.6831	0.0261	-0.2754	0.7637	0.0306	-0.1832	0.5496	-0.1372	-0.1328	0.7270	0.2374
Expert 5	r	0.6018	0.9595	0.6699	-0.8289	0.9887	0.2762	0.4346	0.9162	0.2183	-0.5506	0.9895	0.1937
	p	0.0657	0.0000	0.0341	0.0030	0.0000	0.4398	0.2094	0.0002	0.5445	0.0991	0.0000	0.5919
	z_r	0.6959	1.9391	0.8106	-1.1846	2.5863	0.2836	0.4655	1.5651	0.2219	-0.6193	2.6217	0.1961
	z_u	1.4368	2.6799	1.5514	-0.4438	3.3271	1.0244	1.2064	2.3059	0.9627	0.1215	3.3625	0.9369
	z_l	-0.0449	1.1983	0.0698	-1.9254	1.8455	-0.4572	-0.2753	0.8243	-0.5189	-1.3601	1.8809	-0.5447
	r_u	0.8930	0.9906	0.9140	-0.4168	0.9974	0.7716	0.8356	0.9803	0.7455	0.1209	0.9976	0.7338
	r_l	-0.0448	0.8331	0.0697	-0.9584	0.9513	-0.4278	-0.2685	0.6774	-0.4769	-0.8764	0.9546	-0.4965
Expert 6	r	0.2241	0.9714	0.7505	-0.5617	0.9702	0.1892	0.7503	0.9235	0.9069	-0.5800	0.9888	0.6072
	p	0.5337	0.0000	0.0124	0.0911	0.0000	0.6007	0.0124	0.0001	0.0003	0.0788	0.0000	0.0626
	z_r	0.2279	2.1164	0.9742	-0.6353	2.0949	0.1915	0.9737	1.6124	1.5098	-0.6624	2.5911	0.7045
	z_u	0.9688	2.8572	1.7150	0.1055	2.8357	0.9323	1.7145	2.3532	2.2507	0.0784	3.3319	1.4453
	z_l	-0.5129	1.3756	0.2334	-1.3762	1.3541	-0.5493	0.2329	0.8715	0.7690	-1.4032	1.8503	-0.0363
	r_u	0.7482	0.9934	0.9373	0.1051	0.9931	0.7317	0.9372	0.9821	0.9781	0.0782	0.9975	0.8948
	r_l	-0.4722	0.8800	0.2292	-0.8801	0.8750	-0.5000	0.2287	0.7022	0.6464	-0.8860	0.9518	-0.0363
Expert 7	r	-0.0959	0.9428	0.5955	-0.0634	0.9876	0.6563	0.2386	0.7122	0.2899	-0.0096	0.9921	0.6378
	p	0.7921	0.0000	0.0693	0.8618	0.0000	0.0393	0.5068	0.0208	0.4166	0.9789	0.0000	0.0473
	z_r	-0.0962	1.7623	0.6862	-0.0635	2.5403	0.7863	0.2433	0.8916	0.2984	-0.0096	2.7671	0.7544
	z_u	0.6446	2.5031	1.4270	0.6773	3.2811	1.5271	0.9841	1.6324	1.0392	0.7312	3.5080	1.4952
	z_l	-0.8370	1.0215	-0.0546	-0.8043	1.7995	0.0455	-0.4975	0.1508	-0.4424	-0.7504	2.0263	0.0136
	r_u	0.5680	0.9867	0.8910	0.5898	0.9972	0.9099	0.7548	0.9264	0.7776	0.6238	0.9982	0.9043
	r_l	-0.6842	0.7705	-0.0546	-0.6664	0.9468	0.0454	-0.4602	0.1497	-0.4156	-0.6354	0.9658	0.0136

r ≧ 0.80

0.80 ＞ r ≧ 0.60

r ≦ - 0.80

-0.80 ＜ r ≦ -0.60
